# Role of perioperative parathormone hormone level assay after total thyroidectomy as a predictor of transient and permanent hypocalcemia: Prospective study

**DOI:** 10.1016/j.amsu.2021.102701

**Published:** 2021-08-10

**Authors:** Mohamed S. Essa, Khaled S. Ahmad, Mohammed A. Fadey, Mohamed O. El-shaer, Ahmed M.F. Salama, Mohamed E. Zayed

**Affiliations:** a Department of General Surgery, Faculty of Medicine, Benha University, Benha, Egypt; b Department of General Surgery, Prince Mohammed Bin Abdulaziz Hospital, Riyadh, Saudi Arabia

**Keywords:** *Hypoparathyroidism*, *Hypocalcemia*, *Total thyroidectomy*

## Abstract

**Background:**

The researchers are trying to evaluate the measurement of: Intact parathyroid hormone (iPTH) and serum total calcium (sCa) levels for predicting hypocalcemia after total thyroidectomy (TT).

**Methods:**

The sample of this single center prospective study consists of (100) patients, where (77) females and (23) males with an age range between (28) and (65) (the mean level is, 48.17 ± 6.54). These selected patients underwent total thyroidectomy (TT) in the general surgery department, Benha university hospital from the period of June 2019 to February 2020. Levels of sCa and iPTH were measured aat several times preoperatively, 10 min, 48 h, 3, 6, 9 months, and 1 year after being after gone TT.

**Results:**

Among the entire study sample, 23 patients (23%) developed transient hypoparathyroidism and hypocalcemia (˂8.5 mg/dl), none of them developed permanent hypoparathyroidism and hypocalcemia. The cut-off point of PTH has been 10 min after TT was at 23 pg/mL as it was the best compromise between sensitivity and specificity for predicting hypocalcaemia. It has been found that Patients who have a PTH greater than 23 pg/mL can be discharged safely after 24 h. Patients who have PTH of less than 23 pg/mL were observed for an additional 24 h, and the study found that timely treatment initiation is recommended. A PTH ˂ 10 pg/mL measured at 48 h after surgery had a sensitivity, specificity as well as an accuracy of 100%, for predicting hypocalcemia after TT. The accuracy of a single PTH concentration at 48 h was useful for predicting hypocalcemia [Area under receiver–operator characteristic curve (AUC) 1; confidence interval (CI), 95%, 0.85–0.94].

**Conclusion:**

Patients with iPTH ˂ 10 pg/mL, and sCa levels ˂ 7.4 mg/dL are at higher risk of developing hypoparathyroidism and hypocalcemia after TT.

## Highlights

• Incidence of Post-thyroidectomy transient hypoparathyroidism ranges between 0.3 and 49%, while permanent hypoparathyroidism is 13%.
• Incidence of hypocalcemia following total thyroidectomy ranging between 3% and 40% and is usually transient, while permanent hypocalcemia is ≤ 2%.
• Post-thyroidectomy PTH accurately predict hypocalcemia.

## 1 Introduction

Thyroid surgery can result in serious morbidities such as recurrent laryngeal nerve (RLN) injury, leading to transient or permanent vocal cord paralysis and hematoma that may result in airway compromise. Hypocalcemia is the most common complication after thyroidectomy, which may be temporary or permanent [[1], [2], [3], [4]].

Hypocalcemia after thyroid surgery occurs for several reasons, such as removing the parathyroid gland and injury to its blood supply, leading to transient or permanent hypoparathyroidism. Furthermore, deficiency of vitamin D, hungry bone syndrome, acute rising in serum calcitonin level, hypocalcemia induced by postoperative alkalosis due to hyperventilation from postoperative pain, and postoperative dilutional hypocalcemia can all lead to or exacerbate post-thyroidectomy hypocalcemia [[5], [6], [7], [8], [9], [10]].

Post-thyroidectomy incidence of transient hypoparathyroidism ranges between 0.3 and 49%, whereas permanent hypoparathyroidism is up to 13% [11]. Intraoperative PTH assay has been suggested to have value in predicting and identifying the patients being at risk of post-thyroidectomy hypoparathyroidism and hypocalcemia. Furthermore, intraoperative PTH results can affect postoperative management, such as patients who need early calcium supplementation and eligible for early safe discharge [12,13].

Post-thyroidectomy PTH predicts hypocalcemia accurately. Hypocalcemia is less likely in the presence of a normal PTH level, so PTH can facilitate the patient's discharge within 24 h. Furthermore, PTH levels can be followed to guide early postoperative treatment with calcium and/or cholecalciferol supplements to decrease the incidence and severity of hypocalcemia. Measurement of PTH at any time from 10 min to several hours after thyroidectomy will accurately predict postoperative hypocalcemia. Some surgeons advise oral calcium supplementation routinely to all patients who underwent TT to prevent hypocalcemia [11,14].

The high incidence of post-thyroidectomy hypoparathyroidism and the fact that it is associated with a considerable morbidity rate results in sustained efforts for reliable markers to be found out for prediction of hypoparathyroidism. Many studies demonstrate that PTH is a highly sensitive indicator, with high specificity to predict hypocalcemia after TT [15]. Our study aims to find out if an early reduction of PTH after TT can predict hypocalcemia.

### 1.1 Patients and methods

#### 1.1.1 Study design, setting and period

This is a prospective study of 100 new consecutive patients undergoing TT with preoperative and postoperative time-serial analysis of total calcium and PTH levels, it is implemented from June 2019 to February 2020 in the general surgery department, Benha university hospital.

Inclusion criteria:

1 Benign multinodular goiter (37%).
2 Graves' disease (20%).
3 Papillary thyroid cancer (PTC) (16%).
4 Follicular thyroid cancer (10%).
5 Recurrent goiter (10%).
6 Thyroiditis (7%).

#### 1.1.2 Exclusion criteria

- Patients with concomitant primary hyperparathyroidism.

After obtaining the approval of the Institutional ethical committee's study proposal (IRB No: RC.December 1, 2020)in the form of a written informed consent from the patients for purpose of participation in this study, patients were fully informed about the hazards and benefits of the surgery. The sample size of the study was calculated using online software (https://clincalc.com/stats/samplesize.aspx). The current study has been reported in line with the STROCSS criteria [16]. Registration unique identifying number (UIN): 6941 (https://www.researchregistry.com/browse-the-registry#home/).

Therefore, all selected patients underwent the following:

A-Preoperative assessment including
  1 Full detailed history, including any history suggestive of hypocalcemia.
  2 General and local physical examination.
  3 Laboratory investigation includes routine preoperative assessment, free T3, T4, thyroid-stimulating hormone (TSH), total and ionized serum calcium, PTH, creatinine, and serum albumin. At the time of surgery. It has been found out that renal panel and albumin were normal in all patients
  4 Radiological assessment has been conducted, including neck ultrasonography (U/S) and thyroid scan for those with low TSH.
  5 Indirect laryngoscopy.
B-Operative technique
  1 Under general anesthesia, the patient was placed in a supine position with the neck fully extended. A low collar incision was made and carried down through the subcutaneous tissue and platysma muscle.
  2 Creation of subplatysmal flaps
  3-separation of strap muscles
  3 Ligation of the middle thyroid vein.
  4 Ligation of superior thyroid artery and external branch of SLN identified.
  5 Ligation of branches of the inferior thyroid artery and RLN were identified.
  6 The parathyroid glands were identified, and effort was made to preserve each with an adequate blood supply while moving the gland away from the thyroid lobe.
  7 The thyroid lobe was then removed from its attachments to the trachea by dividing Berry's ligament.
  8 The other thyroid lobe was removed in a similar fashion.
  9 Careful hemostasis was ensured.
  10 A small suction drain was inserted through a small stab wound; it was generally removed after 24 h.
  11 Closure of strap muscles, platysma, and skin.
C- Measurement of sCa and iPTH levels.

A preoperative blood sample was drawn one day before TT for baseline measurement of total sCa and PTH levels. Postoperatively time-serial serum Ca levels were measured with an automated colorimetric method at: 10 min, 48 h, 3, 6, 9 months and one year after thyroidectomy. Besides, serum iPTH levels were measured with the two-site chemiluminescent enzyme-labeled immunometric assay one day before operation, 10 min, 48 h, 3-6-9 months, and a year after surgery. The reference ranges of serum Ca and PTH were 8.5–10.5 mg/dl and 15–65 pg/mL, respectively. The percentage change in Ca was defined as postoperative difference between the first two calcium levels (10 min and 48 h). In addition, the percentage change in PTH has been the difference between preoperative and postoperative PTH values.

D-Postoperative Management:

The study protocol required admission of all patients for at least 3 days postoperative for blood tests. All patients were clinically assessed for the manifestation of hypocalcemia. Hypocalcemia defined as a symptomatic patient or Ca level ˂ 8.5 mg/dl during the hospital stay or at any time after discharge from hospital. Symptoms of hypocalcemia either mild: (easy fatigability, generalized weakness, perioral numbness, numbness at the tips of fingers and toes, and positive Chvostek's or Trousseau's signs) or severe: (carpopedal spasm, convulsions, and laryngospasm).Patient with manifestation of hypocalcemia were treated by intravenous (IV) infusion of calcium gluconate until the improvement of symptoms, this is followed by oral supplementation of calcium and active form vitamin D until the symptoms disappeared totally. Patients were discharged with follow up in outpatient clinic at 3, 6, 9 months and 1 year for measurement of calcium and PTH.

### 1.2 Endpoints

#### 1.2.1 Primary endpoint

Detection of the incidence of hypocalcaemia, variation in perioperative calcium level, variation in perioperative PTH level and cut off level of PTH as a predictor of hypocalcaemia after TT.

#### 1.2.2 Secondary endpoint

An evaluation of predictive factors of hypocalcaemia after TT.

#### 1.2.3 Statistical analysis

Data were analyzed by applying Statistical Program for Social Science (SPSS) version 20.0. Quantitative data were expressed as a mean; it was expressed as frequency and percentage. Independent-samples *t*-test of significance was used when comparing two means. A one-way analysis of variance (ANOVA) applied when comparing more than two means. Post Hoc test: Least Significant Difference (LSD) was used for multiple comparisons among different variables. The confidence interval was set to (95%), whereas the margin of error accepted was set to (5%). The p-value was considered significant if ≤ 0.05.

## 2 Results

This study consists of100 patients (77 women, 23 men) and underwent TT. Demographic characteristics of the patients are summarized and described in Table 1. All patients were normocalcemic preoperatively, none of them developed complications related to surgery and no patients missed follow up in outpatient clinic.

**Table 1:** Demographic characteristics of the patients.

Variable		Total number Of patients (100)
Age, range, (Mean ± SD)		28-65 (48.17 ± 6.54)
**Sex**	**Male**	23 (23%
	**Female**	77 (77%)
**Post-menopausal**		43 (43%)
**Indications of thyroidectomy**	**PTC**	16 (16%)
	**FTC**	10 (10%)
	**Benign multinodular goiter**	37 (37%)
	**Recurrent goiter**	10 (10%)
	**Thyroiditis**	7 (7%)
	**Thyrotoxicosis**	20 (20%)
**Comorbidities**	**Diabetes Mellitus**	31 (31%)
	**Hypertension**	42 (42%)
**Number of parathyroid glands identified during operation**	**0**	1 (1%)
	**1**	5 (5%)
	**2**	27 (27%)
	**3**	55 (55%)
	**4**	7 (7%)

Primary endpoint.

There is a statistically significant variation in normocalcemia and hypocalcemia, according to indications of thyroidectomy (malignant versus benign) and operative procedures (lymph node dissection versus non-dissection), particularly the central group. Serum calcium levels continued to decrease to 48 h after surgery in the hypocalcemic group, but not in the normocalcemic group. There is a statistically significant difference between normocalcemic and hypocalcemic groups according to total calcium level after 48 h and 3 months (P = 0.001)[Table 1]. The low calcium levels slowly increased and recovered to a normal level at approximately 3 months in both groups of men and women after operation. The recovery rate of sCa levels was significantly lower in the hypocalcemic group than the normocalcemic group. All patients with hypocalcemia showed a higher drop in calcium level after an operation in comparison to the normocalcemic group.

Serum PTH concentrations in the hypocalcemic group decreased in most patients after 10 min and 48 h after surgery. Then, they were recovered into a normal level after 3 months of operation in all patients of the hypocalcemic group, therefor there is statistically significant variation between normocalcemic and hypocalcemic groups regarding PTH levels from after 10 min to after 3 months (P = 0.001) [Table 2].

**Table 2:** Total calcium levels between normocalcemic and hypocalcemic group.

Total Calcium (mg/dL)	Normocalcemic group (n = 77)	Hypocalcemic group (n = 23)	P value
**Pre-operative**	9.45 ± 0.48	9.39 ± 0.43	0.374
**After 10 min**	9.36 ± 0.38	9.23 ± 0.24	0.492
**After 48 h**	8.85 ± 0.46	6.54 ± 0.52	0.001
**After 3 months**	9.22 ± 0.56	8.67 ± 0.32	0.001
**After 6 months**	9.32 ± 0.39	9.27 ± 0.27	0.269
**After 9 months**	9.38 ± 0.45	9.14 ± 0.29	0.152
**After 1 year**	9.42 ± 0.50	9.29 ± 0.36	0.264

**Fig. 1:**
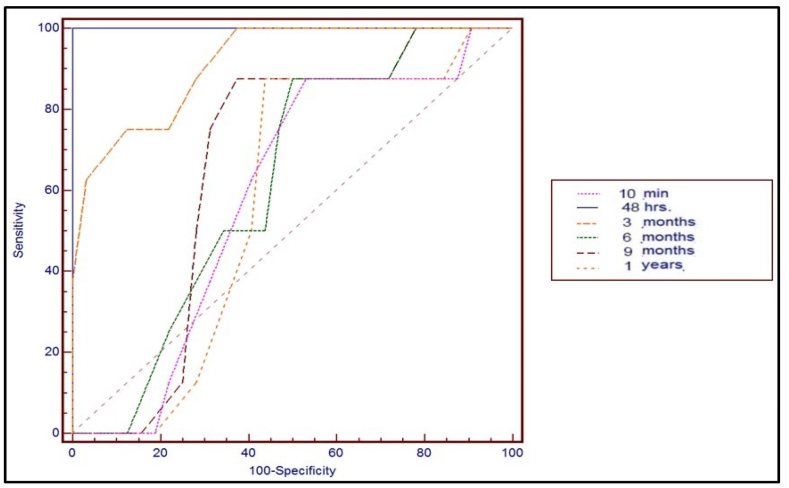
ROC curve for calcium drawn at 10 min, 48 h 3, 6, 9 months and 1 year after TT to predict hypocalcaemia. AUC at 48 h = 1.

**Fig. 2:**
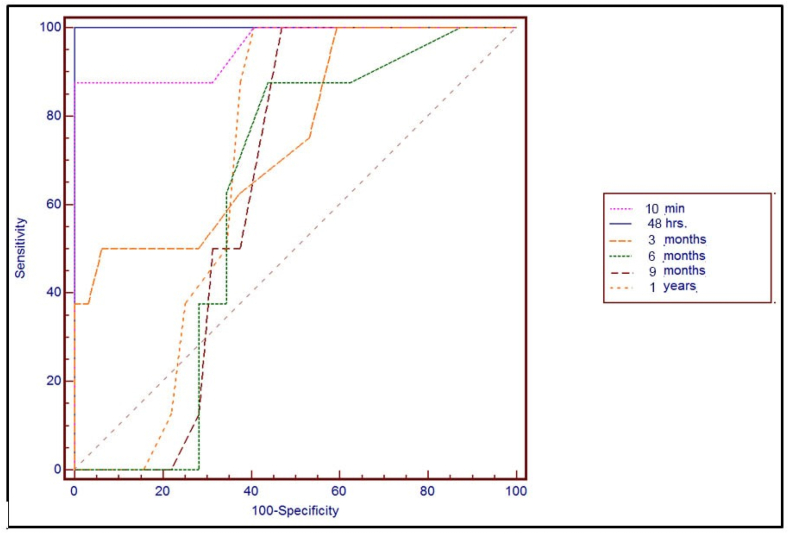
ROC curve for PTH drawn at 10 min, 48 h 3, 6, 9 months and 1 year after TT to predict hypocalcaemia. AUC at 48 h = 1.

Analysis of the test cutoff values indicated much higher sensitivity for the PTH levels at 48 h (100%) than 10 min (89%) after thyroidectomy. The sensitivity of serum calcium levels at the cutoff point increased slightly at 48 h (100%) when compared with those obtained at 10 min after thyroidectomy (88%) [Table 3, Table 4, Table 5]. Receiver operating characteristics (ROC) curve was used to define the best cut off value of Total Calcium, and PTH, The accuracy of a single PTH concentration at 48 h was good for predicting hypocalcemia [AUC 1; CI, 95%, 0.85–0.94] (Fig. 1, Fig. 2). Clinical variables associated with hypocalcemia are illustrated in Table 6.

**Table 3:** PTH level in normocalcemic and hypocalcemic groups.

PTH (pg/ml)	Normocalcemic group (n = 77)	Hypocalcemic group (n = 23)	*P* value
**Pre-Operative**	50.12 ± 4.82	49.11 ± 1.25	0.183
**After 10 min**	41.29 ± 5.57	23.04 ± 6.55	0.001
**After 48 h**	41.12 ± 7.13	7.21 ± 0.63	0.001
**After 3 months**	44.83 ± 5.46	35.76 ± 8.34	0.001
**After 6 months**	48.42 ± 4.74	49.47 ± 2.56	0.641
**After 9 months**	48.57 ± 3.85	50.53 ± 1.89	0.242
**After 1 year**	48.43 ± 4.29	50.79 ± 1.42	0.153

**Table 4:** Accuracy of the cutoff values of calcium in predicting postoperative hypocalcemia.

Calcium Level (mg/dL)	Cut-off value	Sensitivity	Specificity	PPV	NPV	Accuracy
**Pre-operative**	<9.8	90%	56%	33%	96%	57%
**10min after thyroidectomy**	<9.4	88%	51%	30%	95%	58%
**48 h. After surgery**	<7.4	100%	100%	100%	100%	100%
**3 months after surgery**	<8.5	80%	89%	62%	94%	92%
**6 months after surgery**	<9.6	87%	55%	34%	95%	64%
**9 months after surgery**	<9.4	86%	67%	39%	96%	67%
**1 years after surgery**	<9.4	86%	58%	35%	95%	55%

***PPV:*** Positive Predictive Value, ***NPV:*** Negative Predictive Value.

**Table 5:** Accuracy of the cutoff values of PTH in predicting postoperative hypocalcemia.

PTH level (pg/ml)	Cut-off value	Sensitivity	Specificity	PPV	NPV	Accuracy
**Pre-operative**	>50	100%	58%	40%	100%	67%
**10min after thyroidectomy**	<23	89%	100%	100%	98%	95%
**48 h. After surgery**	<10	100%	100%	100%	100%	100%
**3 months after surgery**	<39	54%	95%	69%	87%	77%
**6 months after surgery**	>47	87%	57%	35%	96%	64%
**9 months after surgery**	>48	100%	54%	37%	100%	66%
**1 years after surgery**	>47	100%	60%	39%	100%	73%

**Table 6:** Clinical variables related to hypocalcemia

Variables		Normocalcemic group	Hypocalcemic group	P value
Age, (mean ± SD)		55 ± 11.86	56 ± 12.21	0.23
**Sex**	**Male**	16 (69.6%),	7 (30.4%)	0.34
	**Female**	61 (79.2%),	16 (20.8%)	0.001
**Post-menopause**	**Yes (n = 42)**	29 (69.1%)	13 (30.9%)	0.002
	**No (n = 35)**	25 (71.4%)	10 (28.6%)	0.33
**Hyperthyroidism**	**Yes (n = 24)**	16 (66.7%)	8 (33.3%)	0.001
	**No (n = 76)**	65 (85.5%)	15 (19.7%)	0.42
**Thyroiditis**	**Yes (n = 10)**	6 (60%)	4 (40%)	0.35
	**No (n = 90)**	70 (77.8%)	20 (22.2%)	0.46
**Diabetes**	**Yes (n = 31)**	20 (64.5%)	11 (35.5%)	0.39
	**No (n = 69)**	57 (82.6%)	12 (17.4%)	0.45
**Hypertension**	**Yes (n = 42)**	29 (69.04%)	13 (30.9%)	0.001
	**No (n = 58)**	48 (82.8%)	10 (17.2%)	0.49
**Definitive histological diagnosis**	**Malignant (n = 26)**	15 (57.7%)	11 (42.3%)	0.001
	**Benign (n = 74)**	62 (83.8%)	12 (16.2%)	0.29
**Central LN dissection (n = 26)**	**Unilateral (n = 6)**	3 (11.5%)	3 (11.5%)	0.55
	**Bilateral (n = 20)**	0 (0%)	20 (76.9%)	0.0001
**Total number of parathyroid glands identified during operation**	**0 (n = 1)**	0 (0%)	1 (100%)	0.44
	**1(n = 5)**	2 (40%)	3 (60%)	0.63
	**2 (n = 32)**	22 (68.8%)	10 (31.2%)	0.001
	**3 (n = 55)**	48 (87.3%)	7 (12.7%)	0.32
	**4 (n = 7)**	5 (71.4%)	2 (28.6%)	0.59
**Preoperative calcium levels (mg/dl), ((mean ± SD)**		9.45 ± 0.48	9.39 ± 0.43	0.44
**Preoperative PTH level (pg/ml), (mean ± SD)**		50.12 ± 4.82	49.11 ± 1.25	0.38
**Calcium drop from preoperative to postoperative (48 h)**		0.6 ± 0.02	2.85 ± 0.09	0.001

Secondary endpoint:

Analysis of the factors associated with hypocalcemi:

A univariate and multivariate analysis of the factors associated with hypocalcemia are summarized in Table 7.

**Table 7:** Factors associated with hypocalcemia.

Variables		Odds ratio	95% CI
**Univariate**	**Age ˃55**	0.531	0.266–1.008
	**Female gender**	0.503	0.263–0.874
	**Postmenopause**	0.512	0.323–0.651
	**Hypertension**	0.462	0.342–0.759
	**Diabetes mellitus**	0.422	0.551–0.741
	**Hyperthyroidism**	0.359	0.438–0.871
	**Thyroid cancer**	0.419	0.487–0.776
	**Bilateral central LN dissection**	0.532	0.502–0.864
	**Preoperative calcium**	0.442	0.567–0.797
	**Preoperative PTH**	0.443	0.541–0.679
	**≤ 2 parathyroid identified intraoperativly**	0.359	0.493–0.571
	**Calcium drop from preoperative to postoperative (48 h)**	0.339	0.473–0.582
**Multivariate**	**Age ˃55**	0.642	0.352–1.083
	**Female gender**	0.663	0.357–0.767
	**Hyperthyroidism**	0.721	0.554–0.962
	**Thyroid cancer**	0.614	0.589–0.862
	**Bilateral central LN dissection**	0.543	0.649–0.817
	**≤ 2 parathyroid identified intraoperativly**	0.431	0.651–0.733
	**Postoperative PTH**	0.437	0.663–0.854
	**Calcium drop from preoperative to postoperative (48 h)**	0.445	0.599–0.752

**CI:** Confidence Interval.

## 3 Discussion

Hypocalcemia, after TT, results from either a removal of the parathyroid glands or damage to their blood supply. Incidence of hypocalcemia following TT ranging between (3%) and (40%)is usually transient. The incidence of permanent hypocalcemia in most centers with thyroid surgery experience is ≤ 2% [[17], [18], [19], [20], [21]]. Throughout the current study, the incidence of hypocalcemia was 23%, all were transient.

Monitoring of sCa levels is commonly used to detect post-thyroidectomy hypocalcemia. However, multiple blood samplings to at least the morning following surgery are required. Accordingly, early detection of hypocalcemia after thyroidectomy within the same day of operation is almost impossible due to the fact that PTH is well known to be a useful indicator of impending post-thyroidectomy hypocalcemia, rapid PTH assay has been used to measure intraoperative PTH levels [21].

Early attempts to detect patients at higher risk for post-thyroidectomy hypocalcemia were primarily dealt with to detect the change in sequential calcium level overy time as well as calculating the slope to predict the risk of hypocalcemia. The main concern with this approach was that levels had to be taken over 8 h to be predictive. There was no significant correlation seen with early calcium levels, limiting the use of calcium slopes in facilitating early postoperative hospital discharge [[22], [23], [24]].

Within the current study, 10 min PTH measurement post-thyroidectomy can be used as an indicator of hypocalcemia with (95%) accuracy, which avoids postoperative calcium supplementation and favors early postoperative hospital discharge. A study reported by **Lang et al,** showed that PTH sensitivity and specificity on skin closure were 82.4 and (95.0%), respectively**.** In the current research, 10 min PTH level post-thyroidectomy had a sensitivity of (89%) and a specificity of (100%). In the study of **Lang et al.**, PTH level measurement was withdrawn at the time of the closure of the skin (PTH-SC) because the patient was still anesthetized. So, PTH level results would be ready sooner to help ambulatory surgery. Furthermore, PTH-SC might be a more precise and reliable marker in predicting postoperative hypocalcemia than serial Ca level monitoring; it might also abolish the need for multiple withdrawal of blood samples during hospital stay overnight. In **Lang et al.'s** characteristic curve (AUC), there are different predictors such as sCa slopes, preoperative Ca, PTH-SC, PTH on the next day (PTH-D1), and a combination of preoperative Ca and PTH-SC score that are used for prediction for post-thyroidectomy hypocalcemia. The best cutoff values for PTH-SC and PTH-D1 were 1.0 pmol/L [20].

**Noordzij et al.,** reported that a single PTH threshold (65% decrease in comparison to the level before surgery) measured 6 h post-thyroidectomy had a sensitivity and specificity of 96.4% and 91.4%, respectively in the prediction of hypocalcemia after operation**.** He also demonstrated that if the surgeon's willingness to decrease the risk of subsequent hypocalcemia after the patient was discharged or to avoid unnecessarily calcium supplementation in a patient who is not going to become hypocalcemic; the following 2 cutoff values could be used, the one to 2-h postoperative values (cutoffs of 50% and 90%). In this approach, the patient can be discharged early only if the PTH reduction was ≤50%. Furthermore, only patients with a >90% reduction of PTH should receive Ca supplementation on the operation day. By using these 2 cutoffs, both sensitivity and specificity would be higher than if a single cutoff was applied. The drawback of this approach is that patients in the middle (with a reduction in percent PTH between 50% and 90%) cannot be discharged early and should have traditional serum calcium level monitoring until their serum calcium leveled off [26].

There are several studies in which PTHevaluated either rapid IOPTH or standard-length PTH assay as a predictor for postoperative hypocalcemia after TT which are summarized in Table 8. Blood supply of the parathyroid glands can be assessed through several methods, including the IOPTH levels routinely obtained at the end of thyroidectomy, visual inspection of vascular pedicles of the glands, and small incision of the glands to access bleeding. Because of PTH's short half-life (2–5 min), low levels of PTH can develop within a few minutes following bilateral thyroid dissection. So, the IOPTH level measured immediately after thyroidectomy can be used to predict hypocalcemia [21,29].

**Table 8:** Description of studies regarding PTH assay.

Author (year)	Study type	N	Incidence of hypocalcemia	Method ofPTH assay, (type, reference range [pg/mL])	Times PTH checked	PTH level that predict hypocalcemia	Sensitivity	Specificity
**Lang et al (2012)** [20]	Prospective	117	14.5%	Access immunoassay system (Range 1.2–5.7 pmol/L).	PTH-SC PTH-D1	PTH-SC ˂ 1 pmol/L	82.4%	95%
**Sywak et al (2007)** [25]	Prospective Cohort	100	18%	Chemiluminescent assay was used (Immulite 2000 Immuno analyser	Preop., 4 & 23 h after TT	≤3 ng/L at 4 h	71%	94%
**Macleod et al (2006)** [28]	Prospective	60	25%	Immulite (rapid; range 10–72)	Preop, intraop (5 min after TT), 1 h postop	PACU rPTH ˂ 12 pg/mL	100%	92%
**Friedman et al (2005)** [29]	Prospective	23	Not reported	Automated two-site sandwich immunoassay chemiluminescence system	IOPTH, in the recovery room and on postop. days 1, 7, 14, 21, 28, and 56	IOPTH ˂10 pg/mL	88.9%	92.9%
**Payne et al (2005)** [30]	Prospective	70	24%	Roche Elecsys 2010 (rapid; range 10–70)	1, 6, 12, and 20H postop.	≤28 ng/L at 6 h after TT	100%	100%
**Lombardi et al (2004)** [31]	Prospcetive	53	30.2%	Roche Elecsys E170 (not rapid; range 10–65)	Preop, intraop (after TT), 2, 4, 6, 24, and 48H postop	˂10 pg/mL at 4 & 6 h	94%	100%
**Higgins et al (2004)** [32]	Prospective	104	23.1%	Quick- intra-operative intact PTH KIT	Postinduction, postisolation, 5, 10, 20 min after surgery	˃ 75% reduction in PTH at 20 min	Not reported	Not reported
**Warren et al (2004)** [33]	Prospective	27	11%	Immulite (rapid; range 10–72	Preop, intraop (10 min after TT), 1H postop	≤10 pg/mL 1H after TT	Not reported	Not reported
**Lam et al(2003)** [34]	Prospective	40	30%	Immulite (rapid; range 10–72)	Postoperative (1& 6 h)	≤8 pg/Ml 1H after TT	100%	100%
**Richards et al (2003)** [27]	Prospective	30	33.3%	Immulite (rapid; range 10–72)	IOPTH-SC	IOPTH-SC ˂10 pg/mL	80%	100%
**Warren et al (2002)** [35]	Retrospective case review	23	17.4%	Immulite (rapid; range 10–72)	Preop, intraop (10 min after TT), 1H postop	˂15 pg/mL 10 min after TT	Not reported	Not reported
**Lo et al (2002)** [36]	Prospective	100	11%	ICMA	Intraoperative (at 0 & 10 min)	˃ 75% reduction in PTH at 0 and 10 min	100%	72%
**Lindblom et al (2002)** [37]	Prospcetive	38	26.3%	Elecsys 2010 assay (Roche Diagnostics, Mannheim, Germany) (reference range, 1.6–6.9 pmol/L)	1 day preop., after induction of anesthesia, after resection of 1st lobe, afterresection of 2nd lobe, 1–3 days after TT and at 4th week.	IOPTH ˂ 1.6 pmol/L	90%	75%

**IOPTH-SC:** Intraoperative PTH following skin closure.,**ICMA:**Immunochemiluminometric Assay., **PTH-D1:** PTH at first postoperative day 1., **PACU rPTH:** Post Anesthesia Care Unit Rapid PTH.

With regard to our study, cutoff point of PTH 10 min after TT of 23 pg/mL as has been the best compromise between sensitivity and specificity for predicting hypocalcemia. For patients who have a PTH concentration ≥23 pg/mL, discharge was planned at 24 h after surgery. Patients who have PTH concentration ˂ 23 pg/mL were observed for a further day where timely treatment initiation is recommended.

Some surgeons prefer to prescribe calcium to patients with low calcium levels and/or low PTH after TT. In a study of 120 patients who underwent TT, low total (<7.2 mg/dL) or ionized (<1.0 mmol/L) serum calcium level at 16 h after surgery identified 94.5% of patients who required calcium.Another prospective study of 143 patients undergoing TT, the 112 patients who had a PTH-D1 level ≥10 pg/mL were discharged without calcium supplementation; only 10% developed symptoms of hypocalcemia. By contrast, almost 50% of the other 31 patients who had PTH-D1 level <10 pg/mL developed hypocalcemic symptoms inspite of some receiving calcium supplementation [38,39].

Other surgeons prescribe calcium routinely to all patients after thyroidectomy to avoid hypocalcemic symptoms. In a small randomized trial of 79 patients undergoing TT, routine supplementation with oral calcium and vitamin D decreased both the incidence and severity of hypocalcemic symptoms. In a meta-analysis of 15 studies, routine adminstration of calcium and vitamin D3 after TT was associated with a lower risk of both hypocalcemic symptoms (risk difference −0.25, 95% CI -0.32 to −0.18) and biochemical hypocalcemia (risk difference −0.24, 95% CI -0.31 to −0.17) in comparison to treatment based on measured calcium levels [14,40,41].

The current study has some limitations: first of all, it is a single center study in addition to small number of patients.Therefore, more studies including more cases from multiple centers should be conducted in the future.

## 4 Conclusion

Measurement of the PTH level in the early postoperative period after TT accurately predicts if patients are likely to develop hypocalcemia, it allows the timely initiation of calcium supplementation and may reduce the length of postoperative hospital stay as well as decreasing unnecessary blood tests.

## Ethical approval

All procedures performed in studies involving human participants were in accordance with the ethical standards of the institutional and/or national research committee and with the 1964 Helsinki declaration with its later amendments or comparable ethical standards.

## Consent to participate

Informed consent was obtained from all individual participants who took part in the study.

## Consent for publication

A Written consent was obtained for publication of this study.

## Availability of data and materials

The datasets used and/or analyzed during the current study available from the corresponding author on reasonable request.

## Funding

The authors did not receive support from any organization for the submitted work.

## Authors' contributions

Mohamed S. Essa: Study conception, acquisition of data design and Drafting of manuscript.
Khaled S. Ahmad: Literature review and revision of the manuscript.
Mohamed E. Zayed:Acquisition, analysis and interpretation of data.
Ahmed M.F. Salama: Drafting of manuscript and revision of manuscript.
Mohammed A. Fadey: Revision of the manuscript. Mohamed O. El-shaer:Literature review and revision of the manuscript.

## Guarantor

The corresponding author is the guarantor of submission.

Provenance and peer review.

Not commissioned, externally peer-reviewed.

## Declaration of competing interest

**Mohamed S. Essa:** Declares that he has no conflict of interest.

**Khaled S. Ahmad:** Declares that he has no conflict of interest.

**Mohammed A. Fadey:** Declares that he has no conflict of interest.

**Mohamed O. El-shaer:** Declares that he has no conflict of interest.

**Ahmed M.F. Salama:** Declares that he has no conflict of interest.

**Mohamed E. Zayed:** Declares that he has no conflict of interest.

## List of abbreviations

**iPTH**: Intact Parathrmone Hormone
**sCa**: Serum Calcium
**AUC**: Area Under The Curve
**CI**: Confidence Interval
**RLN**: Recurrent Laryngeal Nerve
**PTC**: Papillary Thyroid Cancer
**MNG**: Multinodular Goiter
**U/S**: Ultrasound
**TSH**: Thyroid Stimulating Hormone
**SLN**: Superior Laryngeal Nerve
**ANOVA**: A one-way analysis of variance
**LSD**: Least Significant Difference
**UIN**: Unique Identifying Number
**IOPTH-SC**: Intraoperative PTH following Skin Closure
**ICMA**: Immunochemiluminometric Assay
**PTH-D1**: PTH at first postoperative day
**PACU rPTH**: Post Anesthesia Care Unit Rapid PTH

## Annals of medicine and surgery

The following information is required for submission. Please note that failure to respond to these questions/statements will mean your submission will be returned. If you have nothing to declare in any of these categories then this should be stated.

## Registration of research studies

In accordance with the Declaration of Helsinki 2013, all research involving human participants has to be registered in a publicly accessible database. Please enter the name of the registry and the unique identifying number (UIN) of your study.

You can register any type of research at http://www.researchregistry.com to obtain your UIN if you have not already registered. This is mandatory for human studies only. Trials and certain observational research can also be registered elsewhere such as: ClinicalTrials.gov or ISRCTN or numerous other registries.

UIN: 6941.
